# A morphometric tool applied to angiogenesis research based on vessel segmentation

**DOI:** 10.1186/1746-1596-8-S1-S20

**Published:** 2013-09-30

**Authors:** Maria-Milagro Fernández-Carrobles, Irene Tadeo, Rosa Noguera, Marcial García-Rojo, Oscar Déniz, Jesús Salido, Gloria Bueno

**Affiliations:** 1 VISILAB, Universidad de Castilla-La Mancha, Spain; 2 Fundación Investigación Clínico de Valencia-Instituto de Investigación Sanitaria INCLIVA, Spain; 3 Laboratorio de Patología Molecular, Dpto. de Patología, Fac. de Medicina y Odontología, Universidad de Valencia, Spain; 4 Dpto. Anatomía Patológica, Hospital General Universitario de Ciudad Real, Spain

## Background

It is now widely accepted that tumor growth and metastasis are angiogenesis and lymphangiogenesis-dependent providing novel therapeutic targets in malignant disease [1-3]. A common feature of the tumor vessels studies is that the investigators focus on microvessel density overlooking other parameters that might be significant, such as the size and shape of the microvessels [4]. In many aspects, tumor vessels are different from normal vessels [5,6]. Studies have revealed the importance of the size and shape of blood vessels in, for example laryngeal tumors [7].

Our aim is to develop a morphometric tool able to perform an easy segmentation of blood and lymphatic vessels to study vascularization following the hypothesis that tumor prognosis may not only be influenced by microvascular density but also by the shape and size of the vessels. To this end a segmentation algorithm based on two complementary methodologies have been developed to segment close and open vessels.

## Material and methods

A dataset of six core images from the Department of Pathology, University of Valencia (DP. UV) was used. They were extracted from TMA scanned slides (Aperio ScanScope XT) at 40x; five images stained with IHC technique against D2-40 and one image from a TMA previously stained with anti-CD34 antibody. Histologic sections comprise two types of vessels: vessels with unquestionable lumen and vessels without evident lumen. Those with defined vascular lumen can be closed or opened, depending on the continuity of the stained endothelial cells in the perimeter.

The images selected to develop the tool contain from low to high number of vessels of both types (from 2 to 600 vessels/image). In the case of closed vessels with and without lumen we can readily calculate their morphometric measurements. The challenge appears when the vascular lumen are not closed or are vaguely stained. In this case a radial algorithm is required.

The algorithm developed by the authors for the segmentation of the vessels, called AngioPath, consists of two parts: a) based on colour segmentation and b) based on the radial distribution of the vessel contour pixels. This algorithm has been compared with other free available algorithm call Caiman [8].

The methods applied for the development of AngioPath is described as follows:

### a) Segmentation based on HSV colour model

Closed vessels with and/or without obvious lumen can be detected through their continuous brown colour. The algorithm proceeds as follows:

1) Conversion of the RGB TMA image to HSV colour model. This permits the segmentation of the brown colour enabling the extraction of the vessel contours.

2) Extraction of the *S* and *H* channels from HSV image. S channel contains most brown shades and ground staining although it is not sufficient. For this reason H channel is also used.

3) Application of a binary inverted thresholding to *S* channel image and of a binary thresholding to H channel image with thresholds of 30 and 20 respectively.

4) Application of a logical *OR* operator to both images. This operation segments brown colour and erases the rest of the colours.

5) Application of a logical *NOT* operator in order to invert the image.

6) Elimination of small artifacts of the input image and joining nearby structures. Erode and a dilate operations of 2 and 4 iterations respectively are performed in the image.

7) Application of a contour-finding operator. This is used to detect the vessel contour pixels that divide each segment of the image, allowing storage through sequences and individual manipulation. This is applied to images created by a binary thresholding or a Canny operator.

8) Discard false positives such as macrophages, which could be detected as small vessels. Only the contours larger than 6 pixels overall size or with a width and height larger than 20 pixels are selected. These valid contours are the valid vessels.

9) Extraction of morphometric measurements (table 1).

**Table 1:** Morphometric Measures.

Measure Name	Meaning	Units
**Position**	x-y coordinates	pixels
**Area**	vessel contour area	physical units (µm)
**Size**	vessel width and height	physical units (µm)
**Vascular Density**		
**Aspect**		
**Roundness**		
**Perimeter Ratio**		

(*) Axes correspond to the best fitting ellipsoid of the vessel contour.

10) Storage of all data measurements in an Excel format file. The final image is saved with the vessel contours in a TIFF format file.

### b) Radial distribution of the vessel contour pixels

This algorithm finds the vascular lumen and their brown endothelial surrounding cells. The unconnected parts are then joined together. Once the open vessels are closed, morphometric measurements are calculated. The algorithm proceeds as follows:

1) Extraction of the green channel from the RGB image. It helps to better distinguish the different vascular lumens. The use of a single channel reduces the computational time and the RAM memory used to process images.

2) Application of a binary thresholding to extract vascular lumens from the image. The threshold value was set to 236.

3) Application of a combination of erode (3 iterations) and dilate (2 iterations) operations to join and remove large and small image structures from the previous step.

4) Filling of those closed contours having internal holes smaller than a minimum size (400 pixels).

5) Performing a second erosion of one iteration which increases the space between the vascular lumens and the vessel membrane.

6) Computing the normal direction of each point of the border of a potential vascular lumen using that direction to check if there are some vessel contour pixels near that point. Vessels are considered as valid depending on the ratio of checked points that are actually membrane vessel points. The ratio is also adjusted depending on the size of the vascular lumen contour.

7) Application of a contour-finding operator to find the vessel contours of the radial algorithm.

8) , 9) and 10) As with the other algorithm, removal of small artifacts, extraction of measurements for each valid vessel and storage of data measurements as well as the final image with the vessel contours.

**Figure 1:**
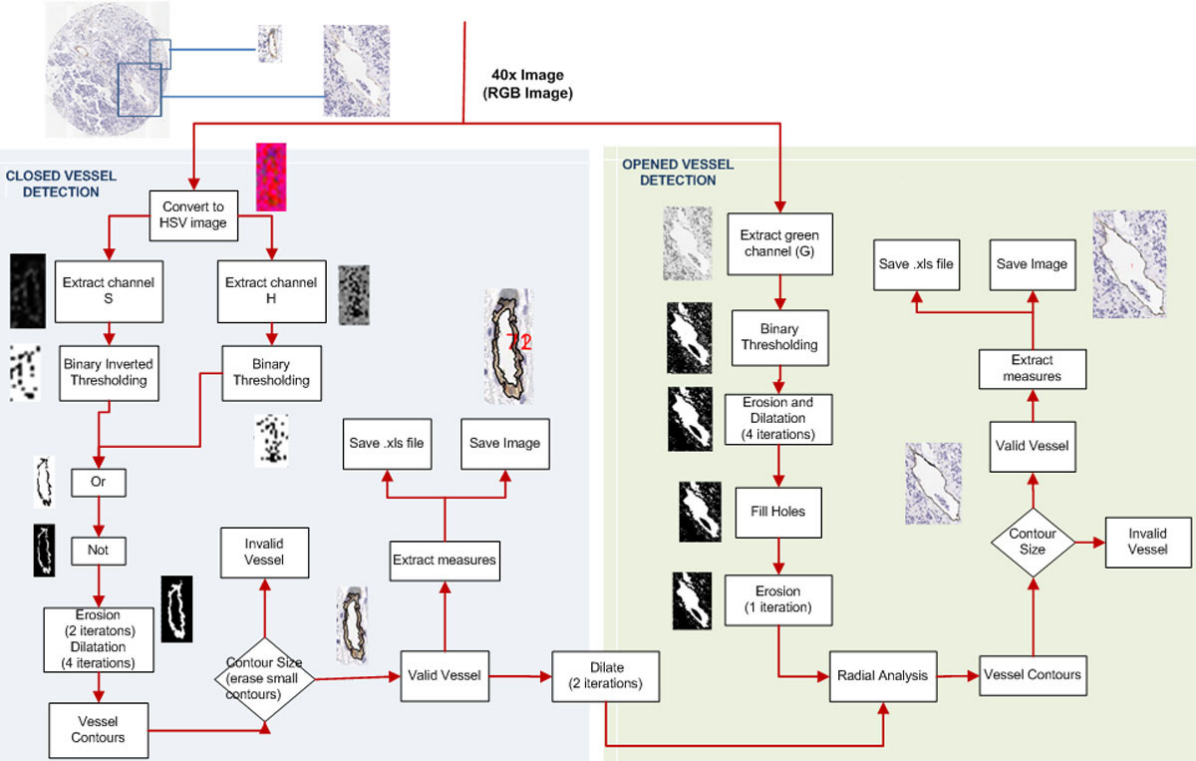
**Vessel segmentation process.** Right panel) segmentation based on HSV colour model and left panel) radial algorithm for detecting open vessels.

The algorithm developed is illustrated in figure 1. The morphometric measurements calculated for each vessel are indicated in table 1. The parameter position enables location of each vessel and thus their elimination in cases of wrong segmentation. These parameters are calculated for the external and internal contour of the vessels.

## Results and discussion

To our knowledge, only two applications provide vessel closing when the whole perimeter of the vessels is not completely stained which could be a basic feature in translational research. Aperio’s application for angiogenesis analysis performs an adequate segmentation of the previously closed vessels but does not provide information about shape [9]. The second algorithm, Caiman, measures shape parameters after closing the vessels but is restricted in the size of the files analyzed (2MB). It shows 96.3 % of the contour pixels correctly detected [8]. AngioPath closes vessels, measures shape parameters, supports any image size and shows an average of 95% sensitivity and 98% specificity (ROC analysis). Among the parameters measured, the shape factors roundness and aspect are calculated in both Caiman and AngioPath algorithms but AngioPath includes perimeter-ratio which indicates the regularity of the contour of the vessels and which we found to be related to clinical-biological features in, at least, neuroblastic tumors (data not shown). AngioPath allows correlation of a measurement with a given vessel and its elimination it if the automatic analysis makes a wrong measurement, thus solving the problem. Caiman lasts an average of 230s for 1,5Mb or 1200x960 pixel images and could not support the image size of our dataset. The average image size of our dataset is 6300x6300 pixels, that is 120MB. AngioPath, takes between 10 to 180 seconds (s) for images with 2 and 600 vessels respectively.

**Figure 2:**
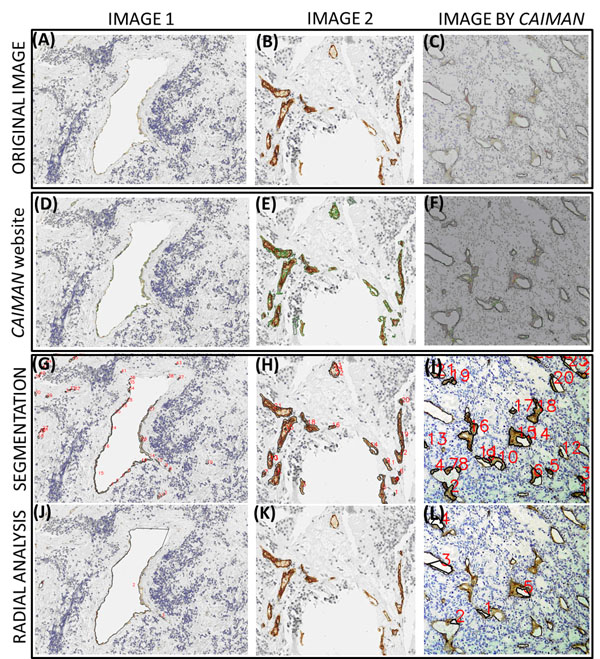
**Comparison of the segmentation quality between the developed tool and CAIMAN application.** 1^st^ row) original images: A and B are images from our database and C is an example image provided by CAIMAN with a less well-lit caption. 2^nd^ row) segmentation performed by CAIMAN (green line) which does not detect small or very large vessels (D) and sometimes divides large vessels into small ones or considers blue areas as vessels (E). The image is well segmented (F). 3^rd^row) result of the HSV segmentation (black line).In our images the morphometric tool makes a specific and good detection of the brown staining (G and H). The fine contour lines of the CAIMAN image have not been detected (I).4^th^row) a result of the radial analysis. The opened contour vessels have been properly closed, including the large vessel (J). The tool detected the absence of opened vessels in the second image (K). Finally, as it does not detect the fine contours in the CAIMAN image, it does not detect the opened vessels (L).

A limited and preliminary analysis of two images from our database and one example image from Caiman was carried out to assess the quality of the segmentation (see figure 2). The study showed a higher fidelity in the segmentation of the vessels for each tool when using its own images. Nevertheless, AngioPath segmentation correlates with the real vascular structures of the Caiman image better than Caiman does with the images from our database. The differences could probably be related to a specific and differently designed brown colour spectrum provided by the stain or the digital image quality.

## Conclusions

The developed tool, AngioPath, is based on the essential property of vessel closing to properly count the number and characterize the size and the shape of blood and lymphatic vessels. In the same way, the set of endothelial cells forming a vessel are then considered together as a single object, making vascular density measurement more accurate.

Although this tool has shown good results in the database tested, it may be improved applying invariant colour analysis techniques to properly segment vessels with different stains as well as improve robustness for segmenting poor quality digital images. This tool it is expected to enable wide studies to be carried out to test if shape and size measurements are important for prognosis.

## List of abbreviations

IHC: Immunohistochemistry; TMA: Tissue microarray

## Competing interests

The authors declare that they have no competing interests

## Authors' contributions

MMF, OD, JS and GB have developed the morphometric tool. IT and RN designed the morphometric tool. MG scanned the images. All authors contributed equally in writing the manuscript.
